# Farmers' suicide in Vidarbha region of Maharashtra state: A myth or reality?

**DOI:** 10.4103/0019-5545.42401

**Published:** 2008

**Authors:** P. B. Behere, A. P. Behere

**Affiliations:** Department of Psychiatry, Mahatma Gandhi Institute of Medical Sciences, Sewagram - 442 102, District Wardha, Maharashtra, India

**Keywords:** Farmers, suicide, psychological autopsy, debt

## Abstract

Incidence of farmers ending their lives in Vidarbha region had hit epidemic proportions recently. We adopted the psychological autopsy approach to offer some insight into the reason why these individuals resorted to such a drastic step. Suicide in farmers is public health problem and we suggested some immediate and serious interventions to prevent suicide.

## INTRODUCTION

*“Jai Jawan , Jai Kisan”* - Lal Bahadur Shastri

This slogan of a visionary prime minister had lost its potential over the time. After the independence, according to Gandhiji's vision of Gram-Swaraj, villages and specially farmers were to be the main focus of any development plan of India. As years passed, by agriculture as an industry lost its importance for policy makers of India. This over the time caused severe distress among the farmers leading to recent dramatic rise in the number of suicides among farmer community. Every day in national newspaper invariably there is news related to farmers' suicides.

India consisting of 16% of world's population sustains only on 2.4% of land resource. Agriculture sector is the only livelihood to the two-third of its population which gives employment to the 57% of work force and is a raw material source to large number of industries. Despite of portrayal of farming as a healthy and happy way of life, agriculture sector experiences one of the highest number of suicides than any other industry. Farmers' suicide is not only reported in Vidarbha region of Maharashtra, but also from Punjab, Uttar Pradesh, Kerala, and Karnataka. Many enquiry commissions were formed and recommendations were implemented especially in Punjab.[1] The problem of suicide is not only reported in India, but also reported in different parts of the world like England and Wales.[2]

In 1990s, India woke-up to a spate of suicide among farmers community. The first state where suicides were reported was Maharashtra with particular reference to Vidarbha region. A look at the figures given out by State Crime Records Bureau makes it evident that farmers as a professional category is suffering from this problem of high-suicide rates. Approximately 3.4 million cotton farmers occupy the Vidarbha region (includes Akola, Buldana, Washim, Amravati, Nagpur, Chandrapur, Gondia, Bhandara, Yavatmal, Gadchiroli, and Wardha districts) and 95% of them struggle with massive debt, according to the Vidarbha Jan Aandolan Samiti (VJAS; Local Farmers' Support Network).[3] Incidence of farmers ending their lives in this region had hit epidemic like proportions recently.

We have studied the status of farmers' suicide in Vidarbha region on the request of Wardha district administration. We adopted the psychological autopsy approach to offer some insight into the reason why these individuals resorted to such a drastic step.[4]

In India, however, problems in identifying the population base and in the certification of death has meant that the true magnitude of the problem is yet to be realized.[5]

Several studies undertaken in India have revealed the incidence of suicides to vary from 8 to 43 per 100,000 population[5] with a pronounced State-to-State variation, the highest being in Kerala (27 per 100,000) while the lowest is in Manipur (0.02% of total suicides). Due to its medico-legal nature, information on suicides is available from national, state, and city crime record bureaus in various parts of India. Majority suicide studies are based on police records with very few from the hospital records and nil from population settings. Given the inadequacies of police reporting - analysis and misclassification bias (suicides, homicides, and accidental deaths) - the numbers may be an under reporting of the situation.

## FARMERS' SUICIDE

Since long time, Indian farmers have been facing a number of socioeconomic problems, such as harassment by moneylenders, inability to repay debts following crop loss, inability to get medical treatment for the family, etc. The problem is compounded by lack of positive and cooperative support from banks especially in the face of inclement weather and market fluctuations. Economic plight of farmers might be illustrated with the fact that a farmer having as much as 15 acres of land and hence considered a well off farmer in Vidarbha, with an average income of Rs 2700 per acre per annum, had an income just little more than what he would have earned the legal minimum wage for all 365 days of the year.

Agriculture is the main stay of the state of Maharashtra. Total irrigated area which had been used for cultivation is 33,500 sq kilometers. Average annual profit from cultivation in the state of Maharashtra is the lowest of all Indian states, lagging far behind the state with the highest - Jammu and Kashmir (Rs. 4363 vs. Rs. 22,770). The reasons for such a pathetic state of farmers include below average rainfall, heavy load-shedding, lack of small irrigation projects, poverty, pressure of private moneylenders and banks, ignorance of ancillary occupations for raising income, employment problem of the farmers' children, decreasing interest of the young generation in farming, rapid urbanization, apathy and lack of political willpower toward welfare and development of the region, etc. Cumulative effect of all these is evident on the psyche of the people of Vidarbha in general and farmers in particular. Farmers are hence prompted to turn to local moneylenders (sahukars) who charged them a much higher rate of interest. In fact moneylenders proved to be the most common and easy source of loans for the farmer (28.4%) followed by loans procured from relatives (22.93%) while only 3.94% turned to land development banks.

## MAGNITUDE OF THE PROBLEM

In a country of 70 million farmers, it is 10 in every 100,000 farmers committing suicide. This is higher than the total national suicide rate.[6] The number of farmers committing suicide in India is more than twice of the total number of suicides being committed in the top 100 countries on the suicide list! This indeed is worrying factor. The Government's measures including waiving off loans, construction of dams, and other assisting measures have not produced positive results so far.

In India, the national data show that suicide rate was 9.7/lakh population in 1995.[1] The population of Vidarbha is 12 lakhs, so number of suicide should be around 116 per year. But according to Vidarbha Jan Andolan Samiti,[3] suicides in Vidarbha is 600 in 2007 till June, 1065 in 2006, 572 in 2005, 620 in 2004, 170 in 2003, and 122 in 2002. These figures definitely suggest suicide rate in Vidarbha is high since 2002 in comparison to national suicide figure. A total of 7000 farmers have committed suicide during the last 3 years. That is an average of over six farmers committing suicide per day! More than 2190 per year!![7] Farmers' suicides in Vidarbha in the last 3-4 years have already crossed 2500 causing a great anxiety.[8]

Wardha district in particular is also facing this problem with increasing number of claims for government ex gratia grant on steady rise.[9] In 2008 till April alone there were 26 claims, as compared to 29 in 2004, 26 in 2005, 154 in 2006, and 128 in 2007. Subsequently Hon. Prime Minister Manmohan Singh visited Vidarbha and promised a package of Rs. 11,000 crores to be spent by the government in Vidarbha. The families of farmers who had committed suicide were also offered an ex gratia grant to the tune of Rs. 1 lakh by the government. This figure kept on varying, depending on how much pressure the government was facing from the media and the opposition parties for being uncaring toward the farmers' plight.

## WHY FARMERS IN VIDARBHA?

Vidarbha is home for approximately 3.4 million cotton farmers and 95% of these are struggling with the massive debt. Most of the villages in Vidarbha are badly in need of basic social infrastructure like all-weather roads, drinking water, regular electricity, primary health care, and basic education. Majority of suicide cases are from cotton growing areas. The cotton farmers in India paying more prices for inputs like seeds, pesticides, fertilizers, electricity, water, and labor whereas the price of cotton has gone down along with decreased productivity. In contrast to this picture in India, US Government is giving USD 4.0 billion to cotton farmers and asking for further liberalization of cotton trade in USA.

As discussed earlier, some of the important contributing factors for farmers' suicide in this region are:

- absence of adequate social support infrastructure at the level of the village and district,
- uncertainty of agricultural enterprise in the region,
- indebtedness of farmers,
- rising costs of cultivation,
- plummeting prices of farm commodities,
- lack of credit availability for small farmers,
- relative absence of irrigation facilities,
- repeated crop failures,
- dependence on rainfall for farming,
- rural living and easy access to poisons, and
- lack of political will and insight in the region.

In Vidarbha (basically a low rainfall area), the major crop is cotton, jowar (Barley), and pulses and people rely more on dry farming. Irrigated farming is insignificant and seen only in very few pockets where major rivers provide water for the whole year.

With the cumulative effect of these reasons coupled with environmental antagonism and exploitation of the farmers in all sectors made them pessimistic toward life resulting into development of suicidal tendency in them. The remedial measures undertaken by the government did not show any immediate positive effect in the attitude of the farmers. In April 2007, an NGO named Green Earth Social Development Consulting[10] brought out a report after doing an audit of the state and central government relief packages in Vidarbha.

The report's conclusions were:

- Farmers' demands were not taken into count while preparing the relief package. Neither were civil society organizations, local government bodies, panchayats, etc. consulted.
- The relief packages were mostly amalgamations of exiting schemes. Apart from the farmer helpline and the direct financial assistance, there was scarcely any thing new being offered. Pumping extra funds into additional schemes shows that no new idea was applied to solve a situation where existing measures have obviously failed.
- The farmer helpline did not give any substantial help to farmers.
- The basis for selection of beneficiaries under the assistance scheme was not well defined. Also, type of assistance to be given led to problems like a farmer needing a pair of bullocks getting a pump set and vice versa (or a farmer who has no access to water sources being given pump sets).

Awareness regarding the package was also pretty low. The report concluded quite alarmingly that the loan burden of the farmers will double in 2008.

## OUR EXPERIENCE: VERBAL AUTOPSY ON FARMERS SUICIDE IN WARDHA DISTRICT

The Department of Psychiatry, Mahatma Gandhi Institute of Medical Sciences, Sewagram, had conducted a study on farmers who committed suicide between January 2005 to March 2006 in Wardha district (having 11 talukas). Verbal autopsy was implemented as a tool of investigation. It is a recognized vital tool in suicide investigation and planning intervention strategies. A structured enquiry form consisting of 52 questions was developed. It consisted both close and open-ended questions. Family members of farmers or landowners who had committed suicide during the above specified period were interviewed. The interview included a case history taken by trained doctors and it revealed a glimpse into the causes and circumstances behind these deaths. After the interview, the family members were given counseling.[4] Counseling centers are open in few talukas.

## DISCUSSION

Farmers are in severe distress and there is an urgent need for increased public awareness among farmers regarding agricultural policies both financial and those extended by the government. If given an option, 40% farmers said that they would like to quit agriculture and take up some other carrier. It is a complex task and requires more than just throwing money at it. Surely, other measures will be needed to set the rural economy on track. Marketing and storage infrastructure will have to be built. New crop patterns that answer to emerging needs will have to be introduced. Better rural credit delivery system will have to be evolved. Self-help groups need to be encouraged.

Finally, suicide should not be viewed as only mental health problem, which is a common notion among people. The various factors which play are: (1) chronic indebtedness and inability to pay interest accumulated over the years, (2) economic decline leads to complications and family disputes, depression, and alcoholism, etc., (3) compensation following suicide (death) helps family to repay debt, (4) grain drain, and (5) the rising costs of agricultural inputs and falling prices of agricultural produce.

In the Indian situation, the causes are multifactorial, cumulative, repetitive and progressive, leading an individual to a state of helplessness, worthlessness and hopelessness, obviously influenced by his social strengths, and weaknesses along with his mental health status.

### Future Task

Religious leaders have a major role to play in suicide prevention since all religions discourage the act of suicide. In this context, especially support provided by Hindu religious values in India is a strong protection against suicidal behavior. So indeed, it is very difficult and needs courage to commit suicide especially by farmers. Blaming only moneylenders, as if they have become plastic bags of rural economy is not the solution. We need to address actual reasons of suicide. There should be a committee, not only of agriculturists, economists, but also psychiatrists and social workers as well. Forming self-help group in every 4–5 villages will help. Suicide in farmers is a public health problem having no borders. It needs immediate and serious intervention.
